# Editorial: Community series in targeting signalling pathways in inflammatory diseases, volume II

**DOI:** 10.3389/fimmu.2025.1639004

**Published:** 2025-06-27

**Authors:** Mirza S. Baig, Faaiza Siddiqi, Rajat Atre, Suraj P. Parihar, Syed Faisal, Uzma Saqib

**Affiliations:** ^1^ Department of Biosciences and Biomedical Engineering (BSBE), Indian Institute of Technology Indore (IITI), Indore, India; ^2^ Division of Medical Microbiology, Faculty of Health Sciences, University of Cape Town, Cape Town, South Africa; ^3^ National Institute of Animal Biotechnology (NIAB), Hyderabad, India; ^4^ School of Life Sciences, Devi Ahilya Vishwavidyalaya (DAVV), Indore, India

**Keywords:** inflammation, macrophages, cytokines, anti-inflammatory drugs, TIRAP (TIR domain-containing adaptor protein), small-molecule inhibitors, peptide inhibitors

**Figure 1:**
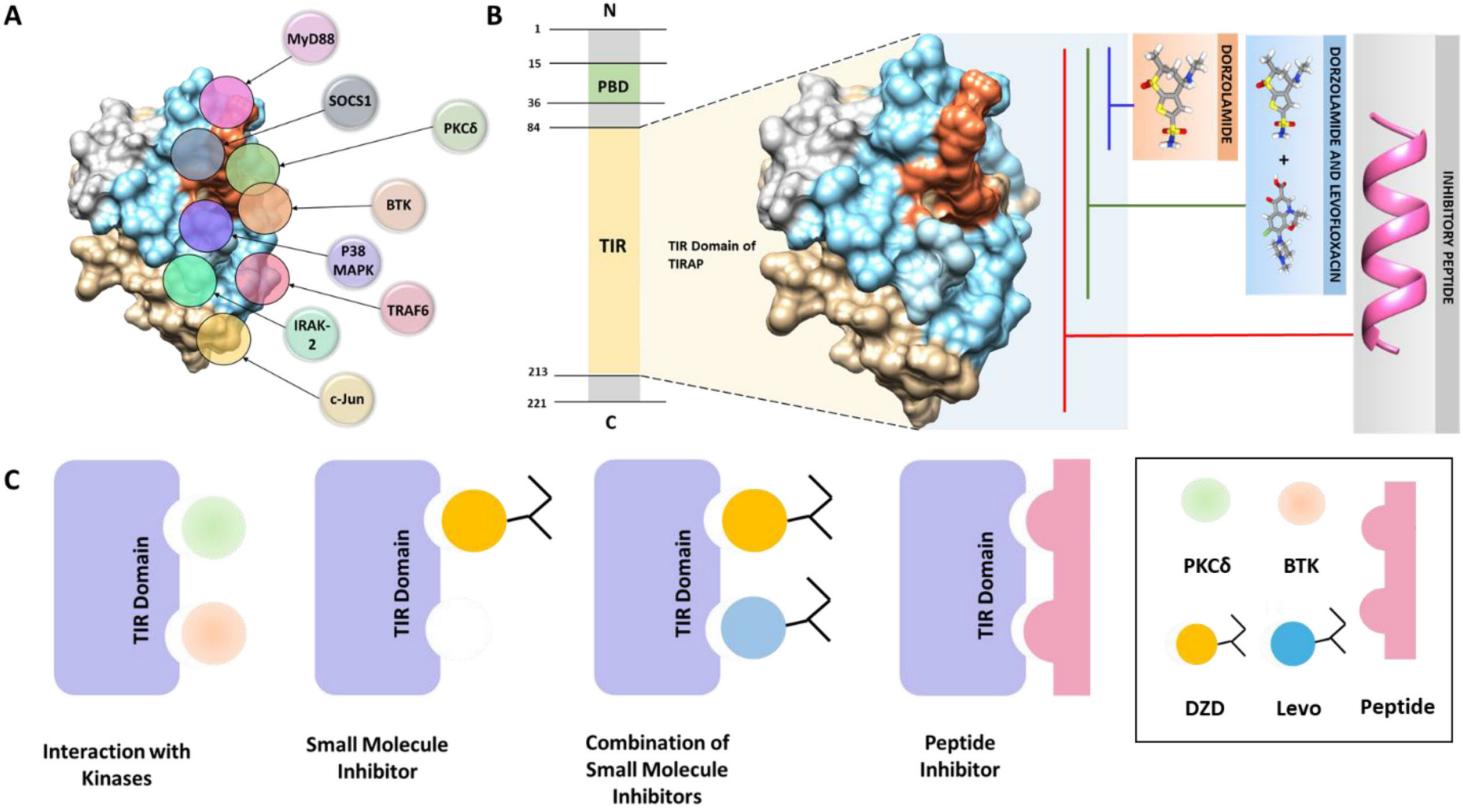
**(A)** Interaction of the TIR domain of TIRAP with various proteins involved in TLR-mediated signaling. **(B)** Strategies for inhibiting the TIR domain of TIRAP, including small molecules, combination therapies, and peptide-based approaches. **(C)** Diagrammatic representation showing inhibition of TIR domain interactions between TIRAP and BTK or PKCδ through targeted small molecules, combination treatments, or peptides.

The Toll-interleukin-1 Receptor (TIR) domain-containing adaptor protein (TIRAP) is a critical intracellular facilitator in immune surveillance that coordinates different signaling pathways. Its utility as a bridging entity, and its versatility in binding with diverse components of Toll-like Receptor (TLR) pathway, has been the subject of several investigations (1). During signal transmission, TIRAP undergoes distinct binding mechanisms and conformational changes leading to differential binding with various intracellular mediators thereby contributing to diverse effects in immunological responses. Among its notable interactions, TIRAP engages with proteins such as MyD88, TRAF6, and IRAK-2, facilitating downstream activation of NF-κB and AP-1 (2). Hence, a convoluted mesh of protein-protein interactions forms the foundation of TIRAP signaling, which is regulated through its TIR domain (2) (**Figure 1A**).

Signaling pathways governed by TIR-domain containing proteins have emerged as a key target for the development of anti-inflammatory therapeutic strategies (3). Dimerization is a central phenomenon required for the functionality of most TIR domains, that span over 200 amino acids and harbor a 14 residues BB loop motif. TIR-mediated signaling mainly relies on the function of the conserved BB loop responsible for signal complex assembly and stabilization (4). TIRAP is a 221 amino acid long protein, which is structured into two main domains namely an N-terminal phosphatidylinositol 4,5-bisphosphate (PIP2) binding domain (PBD) and a C-terminal Toll/interleukin-1 receptor (TIR) domain. TIRAP’s positioning is mediated by Phosphatidylinositol 4-Phosphate 5-Kinase α (PIP5Kα), which generates PIP2, a crucial lipid that serves as a docking site for TIRAP (5, 6). TIRAP’s TIR domain displays structural differences in contrast to the canonic TIR domains. It comprises an extended AB loop that links αA and αB that are developed due to the absence of αB helix classically situated between βB and βC strands (3). These unique structural characteristics of TIRAP have significant implications for immune signaling, as they influence its interactions with other signaling molecules. Consequently, strategies that target and modulate TIRAP-mediated signaling pathways hold promise for the treatment of diseases associated with dysregulated TIR-driven inflammatory responses (3, 7). For example, aberrant TIRAP signaling has been implicated in the pathogenesis of rheumatoid arthritis, where it contributes to destructive inflammation by promoting cytokine production and immune cell activation within affected joints (9).

Mechanistically, TIRAP is known to be activated via a post-translation modification i.e. phosphorylation by kinases namely BTK and PKCδ. Expanding TIR domain targeting through small molecules binding key residues, dual-molecule strategies, or peptide inhibitors spanning the domain, can enhance TIRAP inhibition and disrupt inflammatory signaling (**Figure 1B**). Previously, Rajpoot et al. explored the TIRAP-PKCδ axis and successfully repurposed an FDA approved compound, Dorzolamide (DZD), targeting the interface residues of PKCδ on TIRAP thereby dampening the downstream inflammatory signaling (8). Though DZD attenuated the PKCδ mediated TIRAP activation, BTK-mediated phosphorylation remains an area to explore.

Recently, Baig et al. proposed a combination therapeutic approach for TIRAP-mediated chronic inflammatory septic condition. They addressed two unique aspects of sepsis progression—the destruction of bacteria and the restoration of damaged organs through homeostasis by developing a novel combination of the broad-spectrum antibiotic Levofloxacin and the repurposed anti-inflammatory medication Dorzolamide (LeDoz) (10). Unlike individual drugs that target a single kinase binding site, we discovered that Levofloxacin and DZD interacted with a section inside the binding groove (19 residues) on the TIRAP TIR domain essential for its interaction with not only PKCδ but also with BTK, which are responsible for its activation (10).

Various such alternative modalities have been investigated to silence TIRAP signaling. In one study, molecular-docking and dynamics simulations predicted that the plant alkaloid Narciclasine binds with high affinity to the TIRAP TIR domain as well as other LPS–TLR4-pathway proteins, stabilizing the complexes and thereby suppressing pro-inflammatory signaling (11). In another investigation, Phycocyanin treatment up-regulated miR-3150a-3p, miR-6883-3p and miR-627-5p, which led to depleted TIRAP transcripts and reduced cellular proliferation, thereby establishing a post-transcriptional checkpoint on adaptor availability (12). Interestingly, one study demonstrated that synthetic decoy peptides derived from the TIRAP TIR domain competitively interrupted TIRAP–MyD88 recruitment, abolishing downstream NF-κB activation and highlighting the value of peptide-based blockade of adaptor–adaptor contacts (13). Collectively, these observations indicate that small molecules and miRNA inducers curtail TIRAP through binding or expression control, whereas peptide modalities can potentially better dismantle the protein-interaction surfaces essential for signal propagation. Based on these insights, a therapeutic peptide has been proposed which targets the entire binding pocket in the TIR domain, dampening TIRAP homo-dimerization required for its functionality. THPdb (Therapeutic Peptides and Proteins Database) was screened against the TIRAP TIR domain, identifying a top candidate peptide. Following it's optimization, the peptide exhibited strong binding to TIRAP, interacting with residues 152–193, including the dimerization pocket. Additionally, its binding outside conventional pockets induced structural conformational changes, enhancing its inhibitory effect on TIRAP.

These findings highlight the potential of targeting this domain of TIRAP using small molecules, dual-molecule strategies, or peptide inhibitors as promising approaches to inhibit TIRAP function and disrupt downstream inflammatory signaling pathways. (**Figure 1C**). These strategies could pave the way for novel treatments for chronic inflammatory diseases, providing both structural insights and targeted interventions.
